# Impact of Subolesin and Cystatin Knockdown by RNA Interference in Adult Female *Haemaphysalis longicornis* (Acari: Ixodidae) on Blood Engorgement and Reproduction

**DOI:** 10.3390/insects9020039

**Published:** 2018-04-02

**Authors:** Md. Khalesur Rahman, Mohammad Saiful Islam, Myungjo You

**Affiliations:** 1 Laboratory of Veterinary Parasitology, College of Veterinary Medicine and Bio-Safety Research Centre, Chonbuk National University, Iksan 54596, Korea; khalesurcbnu@gmail.com; 2 Department of Microbiology, Faculty of Veterinary and Animal Science, Hajee Mohammad Danesh Science and Technology University, Dinajpur 5200, Bangladesh; 3 Department of Medicine Surgery & Obstetrics, Faculty of Veterinary and Animal Science, Hajee Mohammad Danesh Science and Technology University, Dinajpur-5200, Bangladesh; saifulhstu@gmail.com

**Keywords:** real-time PCR, RNAi, double-stranded RNA, knockdown, transcript, vaccine

## Abstract

Currently, multi-antigenic vaccine use is the method of choice for the strategic control of ticks. Therefore, determining the efficacy of combined antigens is a promising avenue of research in the development of anti-tick vaccines. The antigen responsible for blood intake and reproduction has proven suitable as a vaccine antigen. It has been shown to silence *Haemaphysalis longicornis* salivary cystatin (HlSC-1) and subolesin by RNA interference. Adult unfed female ticks were injected with double-stranded RNA of (A) subolesin, (B) cystatin, (C) subolesin plus cystatin, and (D) injection buffer, then fed alongside normal unfed males up to spontaneous drop-down. The percentage of knockdowns was determined by real-time polymerase chain reaction. Sixty-three percent and 53% knockdown rates were observed in subolesin and cystatin double-stranded RNA-injected ticks respectively, while 32 and 26% knockdown rates of subolesin and cystatin transcript were observed in subolesin plus cystatin double-stranded RNA-injected ticks. Subolesin and/or cystatin knockdown causes a significant (*p* < 0.05) reduction in tick engorgement, egg mass weight, and egg conversion ratio. Most importantly, combined silencing did not act synergistically, but caused a similarly significant (*p* < 0.05) reduction in tick engorgement, egg mass weight, and egg conversion ratio. Therefore, the elucidation of multiple antigens may be helpful in the future of vaccines.

## 1. Introduction

The hard tick (Ixodidae) is an obligate, blood-sucking ectoparasite that has gained attention due its role in medicine and veterinary science in the transmission of significant pathogens [1]. As an excellent bloodsucker, this arthropod vector transmits a wide variety of infectious agents during the blood-sucking period. Therefore, those employing the concept of Integrated Vector Management should consider interdisciplinary approaches to managing tick populations [2,3]. Control of this dangerous vector is now challenging; tick control mainly depends on the use of acaricides, as well as anti-tick vaccines and tick repellents [4]. Due to the development of resistance and environmental hazards, the use of acaricides is now limited. Recently, nanotechnology has gained attention due to its broad functionality, and the fact that it is generally environmentally sound. Research has shown that zinc oxide nanoparticles from biological sources produce mortality in the freshwater crustacean *Ceriodaphnia cornuta* [5]. Although repellents made from natural products are eco-friendly, many to date have proven to be unable to provide complete protection against tick bites [6,7]. Therefore, the idea of an anti-tick vaccine is a promising approach in the control of ticks and tick-borne diseases. Detection of a suitable antigen is crucial for the development of an anti-tick vaccine. Gene silencing by RNA interference (RNAi) is now a potential tool for detecting the suitability of a candidate vaccine.

Among Ixodidae, *Haemaphysalis longicornis* was reported to transmit a wide variety of pathogens, including *Babesia* spp., *Anaplasma bovis*, *Theileria* spp., *Coxiella burnetii*, *Ehrlichia chaffeensis*, spotted fever group rickettsiae, and severe fever with thrombocytopenia syndrome (SFTS) virus throughout China, Korea, and Japan [8,9,10,11,12,13,14].

Blood is the only nutrient that ticks ingest from vertebrates in order to provide themselves with energy to progress through their life stages. As a three-host tick, *H. longicornis* completes each of its life stages (i.e., larvae, nymph, and adult), with the exception of the egg stage, by successive feeding before dropping to molt for the next stage. Tick salivary glands play a vital role in blood engorgement and the transmission of tick-borne pathogens to vertebrates [11]. During feeding, tick salivary glands secrete a number of bioactive molecules that have immunosuppressive properties, and which are necessary for tick blood engorgement [15]. Therefore, the identification and knockdown of the genes responsible for the secretion of these bioactive molecules is a promising field of research in the development of a tick control strategy.

Cystatin, a large, superfamily natural inhibitor of papain-like cysteine proteases, consists of three groups—type 1 (stefin), type 2 (cystatin), and type 3 (kininogen)—which are widely present in vertebrates, invertebrates, and plants, as well as protozoa [16,17,18]. Type 1 cystatin contains small polypeptides with a lack of the carbohydrate side-chains and disulfide bridges found in other cystatins [19]. Type 2 cystatin is a secretory protein that contains signal peptides; secretory cystatin helps the tick to evade the host’s immune system by processing the antigen-presenting cells (APCs) of the host. Cysteine protease inhibitors known as cystatins have been identified in the sialome of various Ixodid ticks [20]. In *H. longicornis*, three cystatins (Hlcyst-1, 2 and 3) have been characterized [21,22]. Among these, a salivary cystatin, HlSC-1, was identified in the salivary gland of *H. longicornis*, and was found to play a crucial role in the blood-feeding process [23]. One elegant study shows that the knockdown of cystatin by RNAi hampers the tick’s feeding ability and reduces engorgement weight in *Amblyomma americanum* [24]. The other functions of HlSC-1 remain unknown.

A tick protective antigen known as subolesin was recently identified in the *Ixodes scapularis* [25]. Subolesin is an intracellular tick protein ortholog to the akirins of insects and vertebrates that regulates the expression of another gene, and that has an effect on blood engorgement, digestion, reproduction, and development, as well as on innate immune response [26,27,28]. RNAi studies have shown that subolesin gene knockdown in *I. scapularis* affects fertility in males, and promotes a degeneration of the salivary glands, gut, and reproductive and embryonic tissues in females [26,29,30,31].

Injection of subolesin double-stranded RNA (ds RNA) into *Rhipicephalus haemaphysaloides* has been shown to cause a significant decrease in engorgement rate (5.4%), body weight of engorged ticks (206.15 mg), and egg weight (87.84 mg), in comparison with a control group (78.26%, 467.75 mg, and 234.728 mg, respectively) [32]. The researchers showed that recombinant subolesin reduced larval, nymphal, and adult *I. scapularis* infestations (Almazan et al., 2005a; Almazan et al., 2005b). Additionally, Nijhof et al. [31] showed that transovarial knockdown of subolesin in replete females of the *Rhipicephalus microplus* resulted in a silencing in both eggs and larvae. The silencing of subolesin expression with RNAi resulted in a reduction in fertility by 93% and 71% in *Dermacentor variabilis* and *I. scapularis*, respectively [30].

An anti-tick vaccine containing multiple antigens is considered more likely to be effective than one containing a single antigen [33]. Therefore, the researchers focused their study on determining the synergistic effect of subolesin, Bm 86, Bm 91, Rs 86, and voraxin, on different tick species [29,31,34]. Bm 86 homologous to subolesin, a glycoprotein, is the first protective antigen to have been isolated and categorized from the *Boophilus microplus*. Two commercial vaccines have been developed for the control of tick infestations in cattle: TickGARD Plus^®^ in Australia, and Gavac^®^ in Cuba. Both vaccines are based on the recombinant antigen Bm86, located on the mid-gut digestive cell. Bm 91 is a low-abundance glycoprotein located in the salivary glands and the midgut of *B. microplus* (Riding et al., 1994). Bm 91 is a homolog of carboxydipeptidase, and shares many biochemical and enzymatic properties with mammalian angiotensin-converting enzymes. 

In the present study, using RNAi we silenced salivary subolesin and/or cystatin in order to determine their role in feeding and reproduction. This information may help in selecting multiple antigens as candidate vaccines. 

## 2. Materials and Methods

### 2.1. Ticks and Animals

The hard tick, *H. longicornis* (Jeju strain), has been maintained on rabbits at the Laboratory of Veterinary Parasitology, College of Veterinary Medicine and Bio-Safety Research Institute at Chonbuk National University in Iksan, Republic of Korea since 2003 (as described previously) [35]. The animals used in these experiments were treated in accordance with the guidelines of ethical animal research, and were approved for inclusion by the Animal Care and Use Committee of Chonbuk National University (CBNU 2015-003).

### 2.2. Salivary Glands

Twenty unfed, adult female and male ticks of *H. longicornis* were placed on the ears of specific pathogen-free (SPF) New Zealand White rabbits (Samtako, Korea) using a cloth sock attached with tape. After five days of feeding, partially engorged females were removed for salivary gland collection. Ticks were kept for one hour at room temperature for the removal of host tissue. To prevent surface contamination, the ticks were cleaned with distilled water and 70% ethanol. Salivary glands were collected as described elsewhere [36], but with a minor modification: briefly, ticks were attached to a sterile slide (ventral side down) using liquid paraffin. After that, dissection was performed under a dissecting microscope (SMZ-U; Nikon, Tokyo, Japan) using a scalpel fitted with a no. 11 surgical blade. Salivary glands were separated and washed three times with ice-cold 1× phosphate-buffered saline (PBS) to remove midgut contamination, then stored immediately with RNAlater™ (Ambion, Inc., Austin, TX, USA) at −70 °C.

### 2.3. Total RNA Extraction and Synthesis of Complementary DNA from Tick Salivary Glands

Total RNA was extracted from the collected salivary glands using a total RNA extraction kit (RiboEx™) in accordance with the manufacturer’s instructions. The concentration of RNA was determined using a NanoDrop™ 2000 spectrophotometer (Thermo Fisher Scientific, Waltham, MA, USA). The sample was then stored at −70 °C. Complementary DNA (cDNA) was synthesized using a transcriptor first-strand cDNA synthesis kit (Roche Holding AG, Basel, Switzerland) in accordance with the manufacturer’s instructions, using 1 µg of total RNA and an anchored oligo (dT)18 primer.

### 2.4. RT-PCR for Detecting Salivary Cystatin and Subolesin

RT-PCR was performed using BioFACT™ 2X Multi-Star PCR Master Mix with a master cycler gradient (Eppendorf, Hamburg, Germany). For each gene, cDNA was PCR-amplified using a gene-specific primer (Table 1). Actin cDNA was amplified as an internal control using an actin gene-specific primer (Table 1). Oligonucleotide primers were designed based on the sequences of subolesin, cystatin, and actin accession no EU289292.1, AB510962.1, and AY254898.1, respectively. Amplification was performed using a PCR cycle profile, as follows: 95 °C for 15 min, followed by 34 cycles at 95 °C for 20 s, 53 °C for 30 s and 72 °C for one min, with a final extension of five minutes at 72 °C.

### 2.5. Purification of PCR Product and Sequencing

PCR product was visualized by 1% agarose gel electrophoresis. Then, PCR products were purified using an EZ-Pure™ PCR Purification Kit ver. 2 (Enzynomics, Daejeon, Korea) in accordance with the manufacturer’s instructions. Purified PCR products were sent for sequencing. 

Sequence similarities of 97–100% and 97–98% were observed with *H. longicornis* (HlSC-1) cystatin and subolesin respectively, using an NCBI nucleotide blast search (www.ncbi.nih.gov/blast) (data not shown) (GenBank accession No. EU289292.1).

### 2.6. Synthesis of Double-Stranded RNA

The PCR products of subolesin (218 bp) and cystatin (194 bp) were joined to a T7 promoter sequence using T7 promoter-linked (at both the 5′ and 3′ ends) oligonucleotide primers (Table 1). A T7 promoter sequence was added, as described elsewhere [37]. The PCR amplification profile was as follows: 95 °C for 15 min, followed by six cycles at 95 °C for 20 s, 63 °C for 30 s, and 72 °C for one minute, then 28 cycles at 95 °C for 20 s, 77 °C for 30 s, and 72 °C for one minute, with a final extension at 72 °C for five minutes. PCR bands were checked with a 1% agarose gel electrophoresis. PCR products were purified by EZ-Pure™ PCR Purification Kit ver. 2 (Enzynomics, Daejeon, Korea) in accordance with the manufacturer’s instructions. Double-stranded RNA was synthesized from T7 linked DNA using a HiScribe™ T7 High Yield RNA Synthesis Kit (New England Biolabs, Inc., Hitchin, UK) in accordance with the manufacturer’s protocol. The concentration of dsRNA was measured using a NanoDrop™ 2000 spectrophotometer (Thermo Fisher Scientific, Waltham, MA, USA). The sample was then aliquoted and stored at −70 °C until next use. 

### 2.7. Injection of Double-Stranded RNA 

Eighty adult, unfed female ticks were divided into four groups, with each group containing an equal number (20) of ticks. The injection dose for the target gene was selected using methods described in previous research [24,30]. Two groups of ticks were injected with subolesin and cystatin ds RNA respectively (500 ng/tick). The third group was injected with an equal amount of subolesin and cystatin ds RNA mixture, while the negative control (fourth) group was injected with the injection buffer as a control. Ticks were injected with ds RNA as described elsewhere [29,38] using a Hamilton^®^ 33-gauge needle. After injection, the ticks were kept overnight in a 25 °C incubator with high humidity to observe their survival. The injected ticks of each group were mixed with an equal number of male ticks and then placed on the ears of four SPF rabbits. To observe gene silencing, 10 female ticks of each group were collected after five days of feeding. Their salivary glands were subsequently collected, and real-time PCR was performed for gene expression analysis. The rest of the ticks were fed up to spontaneous drop-down. Feeding time, blood engorgement, egg mass weight, and hatching rate were recorded.

### 2.8. Analysis of Gene Silencing at Messenger RNA-Level by Real-Time Polymerase Chain Reaction

Total RNA was collected from the salivary glands of the injected female ticks who had fed for five days, as described above. Real-time PCR was performed to determine the gene expression after knockdown, using a One-step SYBR^®^ PrimeScript™ RT-PCR kit II (Clontech Laboratories, Mountain View, CA, USA) with a Thermal Cycler Dice™ system (Takara, Kyoto, Japan). The primer used for gene expression is cited in Table 1. PCR amplification was carried out in accordance with the manufacturing recommendations. Briefly, PCR amplification was conducted in the following three stages: stage 1 (reverse transcription, 42 °C for five minutes, followed by 95 °C for 10 s); stage 2 (PCR reaction repeats 40 cycles of 95 °C for five seconds, 60 °C for 30 s); and stage 3 (dissociation). Data were normalized with internal control actin and ΔΔ*C*t value, and the percentage of knockdowns was calculated in the same manner as with previous RNAi experiments [32,39].

### 2.9. Statistical Analysis

Statistical analysis was performed with a Student’s *t*-test (unpaired and unequal variances), executed with Graph Pad Prism 5 (GraphPad Software, Inc., La Jolla, CA, USA). Values were represented in the format of mean ± SE. Values for *p* that were 0.05 or less were considered to be statistically significant in comparison with the control group.

## 3. Results

### 3.1. Detection of Salivary Cystatin and Subolesin by Real-Time Polymerase Chain Reaction and Sequencing

**Figure 1:**
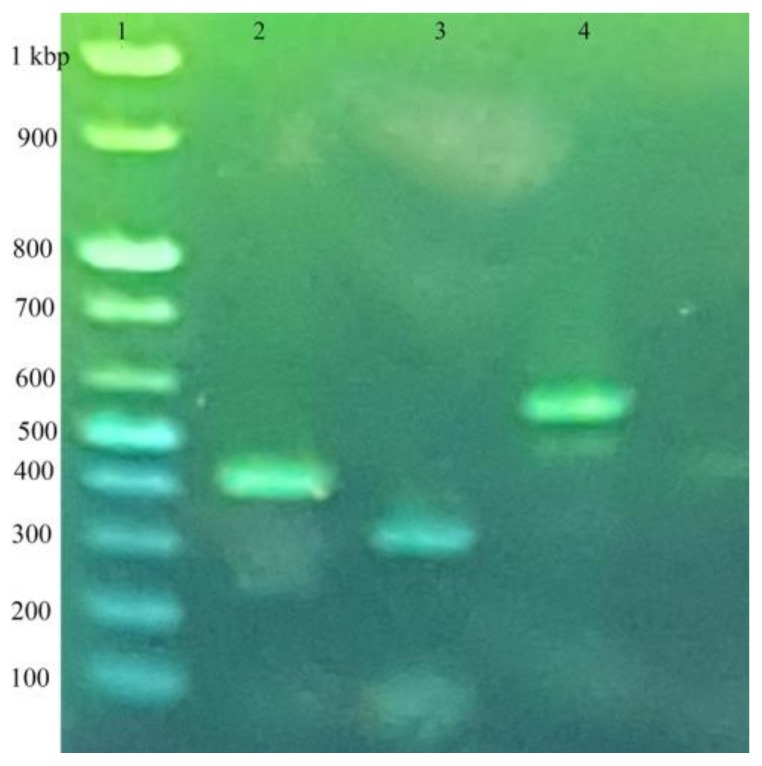
Detection of salivary cystatin (HlSC-1) and subolesin by semi-quantitative RT-PCR. Salivary glands were collected from adult female *H. longicornis* which had been fed for five days. Total RNA was extracted, and a cDNA synthesis was performed. An RT-PCR was performed using gene-specific primers. Lane 1 indicates a 100 bp DNA ladder; lane 2, subolesin 396 bp; lane 3, cystatin 300 bp; and lane 4, actin 540 bp.

The salivary cystatin of *H. longicornis* (HlSC-1) was more abundant in the salivary glands than in the midgut; it plays a crucial role in blood engorgement [23]. Salivary cystatin cDNA encoding 423 bp was identified, as described previously [23]. Cystatin cDNA 300 bp was amplified (Figure 1) with a gene-specific primer (Table 1), and purified PCR products were sequenced. 

Subolesin, a known tick protective antigen, was present in different organs, including the salivary glands and midgut [32]. We amplified the *H. longicornis* subolesin gene encoding 396 bp (Figure 1), as mentioned previously, reference [29] using a gene-specific primer (Table 1).

### 3.2. Silencing of Subolesin and Cystatin by RNA Interference

**Figure 2:**
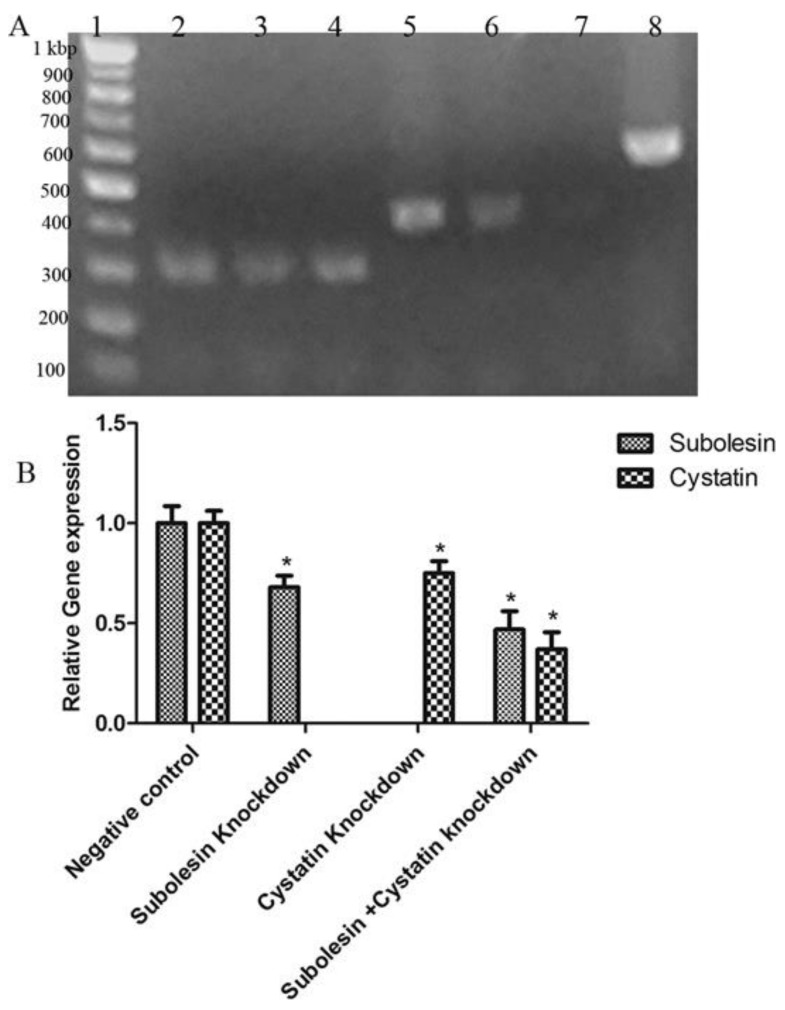
Quantitative and semi-quantitative RT-PCR showing knockdown of subolesin and cystatin by RNAi. (**A**) Semi-quantitative RT-PCR. Lane 1: 100 bp DNA ladder; lanes 2 to:, cystatin from the salivary glands (lane 2 was cystatin from subolesin + cystatin, lane 3 was cystatin from cystatin, and lane 4 was the control group); lanes 5 to 7: subolesin from the salivary glands (lane 5 was the control group, lane 6 was subolesin from subolesin + cystatin, and lane 7 was subolesin from subolesin ds RNA-treated group); and lane 8: actin used as an internal control; (**B**) Quantitative real-time RT-PCR showing subolesin, cystatin, subolesin + cystatin, and negative control ds RNA-treated messenger RNA levels. The data represent mean ± SD, normalized to the actin transcript level. The asterisk (*) denotes a significant difference in comparison with the control group, as determined by a Student’s *t*-test (*p* < 0.05).

Here, we observed gene silencing with both the RT-PCR and the real-time PCR (Figure 2). Cystatin ds RNA-injected ticks showed significantly (*p* < 0.05) decreased transcript levels (63%) after five days of feeding, in comparison with the control group (Figure 2B). Subolesin ds RNA-injected ticks also showed significantly (*p* < 0.05) decreased transcript levels (53%), compared with the control group. It was interesting to observe that the cystatin plus subolesin ds RNA-injected group showed significantly (*p* < 0.05) decreased transcript levels—by 32% and 26% respectively—for subolesin and cystatin (Figure 2B), compared with the control group.

### 3.3. Effects on Feeding Duration and Engorgement

**Figure 3:**
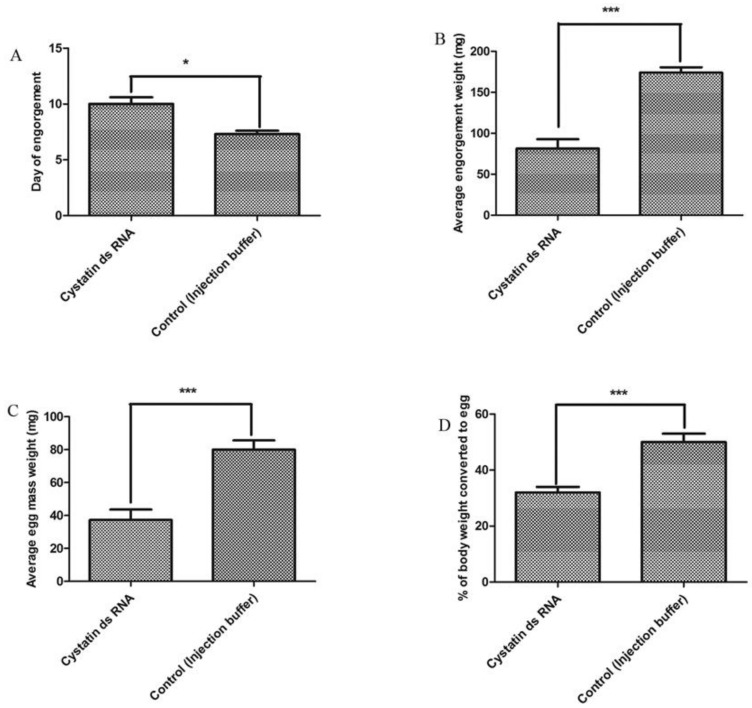
Knockdown effects of cystatin ds RNA on tick engorgement and reproduction. (**A**) Average days of engorgement treated by cystatin ds RNA and the control group (injection buffer); (**B**) average engorgement weight in response to cystatin ds RNA and the control group (injection buffer); (**C**) average egg mass weight in response to cystatin ds RNA and the control group (injection buffer); and (**D**) average egg conversion ratio between cystatin ds RNA and the control group (injection buffer). The asterisk (*) denotes a significant difference compared with the control group as determined by a Student’s *t*-test (* *p* < 0.028; *** *p* < 0.0001).

**Figure 4:**
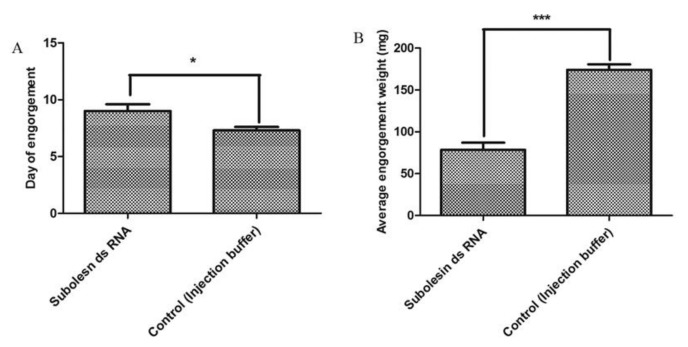
Knockdown effects of subolesin ds RNA on tick engorgement and reproduction. (**A**) Average day of engorgement, and (**B**) data of average engorgement weight between subolesin ds RNA and the control group injected with injection buffer. The asterisk (*) denotes a significant difference compared with the control group as determined by a Student’s *t*-test (* *p* < 0.015; *** *p* < 0.0001).

To verify the effects of subolesin and cystatin knockdown, engorgement weight and feeding duration were analyzed after spontaneous drop-down of the injected ticks. Cystatin knockdown caused a significant (*p* < 0.05) increase in feeding duration (Figure 3A) and retarded engorgement weight, compared with the control group (Figure 3B). The average engorgement weight of cystatin ds RNA-injected ticks was 81.38 mg, while that of the control group was 174.13 mg. Similarly, subolesin knockdown significantly (*p* < 0.05) increased feeding duration (Figure 4A), and retarded engorgement weight, compared with the control group (Figure 4B). The average engorgement weight of subolesin ds RNA-injected ticks was 78.5 mg.

### 3.4. Combined Effects on Feeding Duration and Engorgement

**Figure 5:**
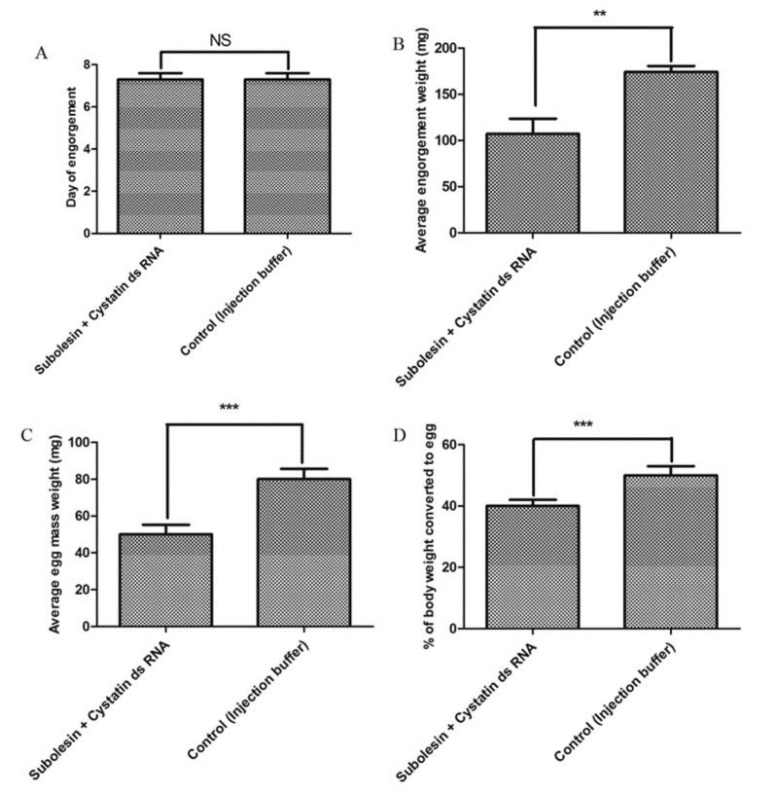
Knockdown effects of subolesin + cystatin ds RNA on tick engorgement and reproduction. (**A**) Average day of engorgement; (**B**) average engorgement weight; (**C**) average egg mass; and (**D**) average egg conversion ratio data were compared between subolesin + cystatin ds RNA and the control group injected with injection buffer. The asterisk (*) denotes a significant difference compared with the control group as determined by a Student’s *t*-test (** *p* < 0.002; *** *p* < 0.001). NS means not significant.

**Figure 6:**
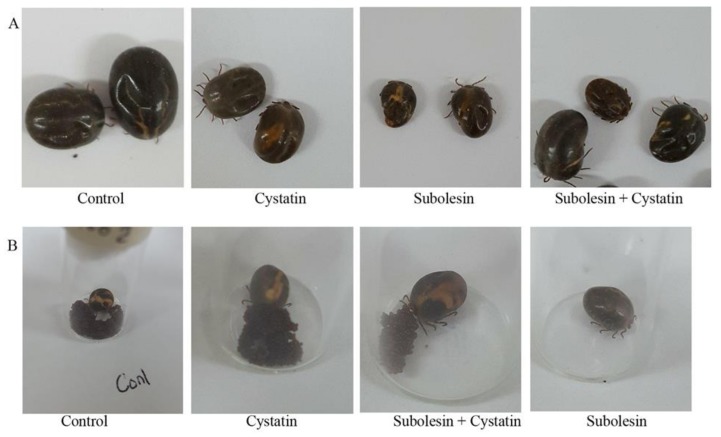
Phenotypic changes of tick engorgement (**A**) and egg masses (**B**). Cystatin, subolesin, and a mixture of subolesin + cystatin ds RNA were injected into unfed female ticks. The control group was injected with an injection buffer. The injected ticks were fed alongside an equal number of males, up to spontaneous drop-down.

To assess the synergistic role of feeding, the mixture of subolesin and cystatin ds RNA was injected into unfed females that were then allowed to feed up to spontaneous drop-down. Feeding duration was similar to that of the control group—seven days on average (Figure 5A). Engorgement weight (average: 107.13 mg) was significantly (*p* < 0.002) reduced in comparison to the control group (average: 174.13 mg) (Figure 5B), but not in comparison to individual gene silencing. Phenotypic changes are illustrated in Figure 6.

### 3.5. Effects on Reproduction

To assess the role of reproduction, egg mass, egg conversion ratio, and hatchability were recorded after spontaneous drop-down of subolesin and cystatin ds RNA-injected ticks. Subolesin ds RNA abrogated egg production, whereas cystatin ds RNA-injected ticks showed significantly (*p* < 0.05) reduced egg mass (average: 37.25 mg) in comparison with that of the control group (average: 79.87 mg) (Figure 3C). The egg conversion ratio was also reduced significantly (*p* < 0.05), compared with the control group (Figure 3D). No differences in hatchability were observed.

### 3.6. Combined Effects on Reproduction

To verify the combined role of reproduction, egg mass, egg conversion ratio, and hatchability were recorded after spontaneous drop-down of subolesin + cystatin ds RNA-injected ticks. Combined silencing was shown to cause a significant (*p* < 0.001) retardation of egg mass and egg conversion ratio (average: 50 mg and 40% respectively), compared with those of the control group, (79.87 mg and 50% respectively) (Figure 5C,D). No differences in hatchability were observed. Therefore, our data indicates that the knockdown of subolesin and/or cystatin plays a significant role in blood feeding and reproduction of *H. longicornis*.

## 4. Discussion

The hard tick *H. longicornis* depends on host blood for survival and reproduction. Tick feeding capability largely depends on its major secretory organ, the salivary glands [40]. Therefore, addressing tick salivary genes is a promising field of research in the development of a tick control strategy. The reverse genetic process RNAi is a powerful approach for the silencing of tick salivary genes [41]. The synergistic effects of tick subolesin, combined with Bm 86, Bm 91, and voraxin silencing, have been reported previously in different tick species [29,34]. Another important salivary molecule, salivary cystatin (HlSC-1), plays a vital role during the early period of blood feeding [23]. A previous study suggests that salivary cystatin may have other functions which are unrelated to blood intake. In *Ornithodoros moubata*, salivary cystatin OmC2 is an inhibitor of cysteine proteases that resembles human cystin C [42]. Therefore, it could be an important immuno-dominant molecule whose inhibitory function might hamper tick feeding and tick-borne disease transmission. In nematodes, cystatins hamper the release of proteases in APCs from the host’s immune system [43]. The silencing of cystatin and the SNARE protein synaptobrevin revealed a significant reduction of body weight in *A. americanum* [24]. The authors report that both cystatin and synaptobrevin are important for blood intake in *A. americanum*. Both subolesin and cystatin have significant roles in blood intake. Therefore, we silenced both of these salivary antigens in order to determine their synergistic role in blood intake, as well as their effects on egg production. In our research, it was clear that cystatin knockdown hampers blood engorgement and subsequent egg production. However, the combined effect of subolesin and cystatin silencing has not yet been studied. Our present study aimed to determine the effects of subolesin and/or cystatin knockdown on feeding and reproduction in the *H. longicornis.*

The silencing of the tick protective antigen subolesin by RNAi resulted in a significant reduction of body weight and egg mass, and increased mortality in *R. microplus*, *I. scapularis*, *R. haemaphysaloides*, *Dermacentor marginatus*, and *D. variabilis* [25,26,30,31,32]. The immunization of mice and rabbits with recombinant subolesin reduced engorgement and egg mass in *R. haemaphysaloides*, indicating their possible future use as part of a cocktail of vaccine antigens [32].

In our present study, the silencing of subolesin by RNAi yielded a significant (*p* < 0.05) reduction of engorgement weight in comparison with that of the control group [44]; importantly, subolesin ds RNA-injected ticks showed abrogated egg production. Similarly, in *I. scapularis*, *A. americanum*, and *D. marginatus* 4D8, ds RNA abrogated egg production [26]. A real-time PCR from the salivary glands of subolesin ds RNA-injected ticks which had been fed for five days showed a reduction of 53% in transcript level (Figure 2). Similarly, a 50 to 90% reduction of cystatin transcript level was also observed in a gene-silencing experiment [24]. 

We detected and purified salivary cystatin for the synthesis of ds RNA. After cystatin ds RNA-injected ticks were fed for five days, they showed a reduction of 53% in transcript level. Resultant significant (*p* < 0.05) reductions of engorgement weight, egg mass, and egg conversion ratio in comparison with the control group were observed. Similarly, 50% knockdown of salivary cystatin caused a significant reduction of tick engorgement in *A. americanum* [24]. 

Currently, multi-antigenic vaccines are preferred over single antigenic vaccines [45,46]. In RNAi experiments, the synergistic effects of silencing multiple genes were determined. In *A. americanum*, a cystatin and synaptobrevin knockdown reduced successful tick feeding [24]. The silencing of subolesin and Bm 86 by ds RNA injection into unfed *B. microplus* females reportedly led to a significant reduction in tick weight and egg/tick mass, and also to an increased tick mortality [31]. In *Rhipicephalus sanguineus*, silencing of Rs 86 and subA caused a reduction in attachment, engorgement, and oviposition [29]. While the injection of subolesin + voraxin ds RNA into *Amblyomma hebraeum* ticks resulted in the silencing of only the subolesin in females which fed together, this was not the case for voraxin [34]. However, combined ds RNA may suppress the function of another gene, and also act synergistically in most cases. As in *Caenorhabditis elegans*, the injection of zfp-1 ds RNA suppressed the lethality produced by mom-2 and hmp-2 ds RNAs [47]. In our research, a combined knockdown did not abrogate egg-laying, but rather, caused a significant retardation of engorgement weight, egg mass, and egg conversion ratio in comparison with the control group (Figure 5). Therefore, our results suggest that subolesin and/or cystatin may be useful as part of a future cocktail of vaccine antigens.

## 5. Conclusions

In the future, combined antigens will be used for controlling ticks and tick-borne diseases. RNAi can be successfully utilized to determine the efficacy of combined gene silencing before vaccination trials. However, it remains a challenge to determine the success of vaccine antigens working together. To our knowledge, the present study is the first to describe the effects of subolesin and/or cystatin knockdown in *H. longicornis.* As multi-antigenic vaccines are more efficient than single-antigenic ones, our findings are expected to lead to subolesin and/or cystatin being considered as vaccine antigens.

## Figures and Tables

**Table 1:** Primers used in this study.

Primer Name	Sequence (5′-3′)	Product Size	Use
HlCF	TTCTAGCAACACGCGTCAAC	300 bp	Cystatin-identifying primer
HlCR	TCACTCCCATTACCCAGAGC		
HlSF	TTAAAGCGGACACACGATTG	396 bp	Subolesin-identifying primer
HlSR	GCTCTCTCGCTCCTTCATCA		
HlAF	CCAACAGGGAGAAGATGACG	540 bp	Actin-identifying primer
HlAR	ACAGGTCCTTACGGATGTCC		
Sub-1 F	TAATACGACTCACTATAGGGTACT ACGTCCCACCGAAGTTGA	218 bp	Subolesin ds RNA synthesis
Sub-1 R	TAATACGACTCACTATAGGGTACT CTGGCGGAAGGTGAACAG		
Sub-2 F	ACGTCCCACCGAAGTTGA	218 bp	Subolesin real-time PCR
Sub-2 R	CTGGCGGAAGGTGAACAG		
Cys-1 F	TAATACGACTCACTATAGGGTACTGACACCAAAACCCTTTGAGC	194 bp	Cystatin ds RNA synthesis
Cys-1 R	TAATACGACTCACTATAGGGTACTGGGAACCTTGTTAGAAGAGC		
Cys-2 F	GACACCAAAACCCTTTGAGC	194 bp	Cystatin real-time PCR
Cys-2 R	GGGAACCTTGTTAGAAGAGC		
Actin-2 F	AGCGTGGCTACTCTTTCACC	229 bp	Actin real-time PCR
Actin-2 R	GATTCCATACCCAGGAACGA		

The underlined bases denote T7 promoter sequences.
